# Interpreting the changeable meaning of hashtags: Toward the theorization of a model

**DOI:** 10.3389/fsoc.2022.1104686

**Published:** 2023-01-16

**Authors:** Gevisa La Rocca, Giovanni Boccia Artieri

**Affiliations:** ^1^Faculty of Human and Social Sciences, Kore University of Enna, Enna, Italy; ^2^Department of Communication Sciences, Humanities and International Studies, University of Urbino Carlo Bo, Urbino, Italy

**Keywords:** agency, cultural morphogenesis, hashtag studies, hashtag activism, Twitter

## Abstract

This study contributes to the international debate on the hashtag's nature and characteristics and attempts to define it as a relational social form affected by morphogenetic–morphostatic processes. To develop this interpretative proposal, this study uses the dimensions of time and agency, drawing on Twitter hashtag studies. Subsequently, the article recalls elements of cultural morphogenesis, traces the points of contact between hashtag studies and cultural morphogenesis, constructs an interpretative proposal of the hashtag as a relational social form, and arrives at the formalization of a model for analyzing the changing meaning of hashtags.

## 1. Introduction

Within communication research, a line of studies has developed over the last 15 years that focuses on hashtags, which can be considered a branch of research with and on big data. In a 2015 book, Rambukkana defines “hashtag publics” as a vector that generates discursive assemblages for techno-social events. The scholar claims that topical hashtags are generated from “nodes in the becoming of distributed discussions in which their very materiality as performative utterances is deeply implicated” (Rambukkana, 2015, p. 3). In this statement he refers to the *On Actor-Network* by Latour (1996), taking up the idea that “the modern understanding of the social sciences as describing some form of pre-existing substance (‘the social') […] drills down into and unearths their other major aspect, which looks at the process and becoming of social forms (Latour, 1996, p. 2; Rambukkana, 2015, p. 1)”. In this study, we keep in mind Rambukkana's position and expand upon it by considering audiences as relational and the hashtag as a relational social form.

Although the hashtag is nothing more than a label (tag) preceded by the hash sign (#), over time it has become much more than a tool for classifying the content of text and images conveyed through the internet, and through social media. From its appearance to today, this sign's meaning, use, and relationship with the social context have undergone a process of transformation that has opened a breach in the in terms of its function, how its use is viewed, and its morphogenetic processes. In hashtag studies, several scholars have asked themselves questions that call into question concepts already analyzed within the social sciences, and especially within the theories that deal with socio-cultural change. In fact, these scholars wonder how to look at the agency of hashtag users who, through their actions over time, reconfigure its meaning (e.g., Yang, 2016). They also question themselves on how to interpret the relationship between hashtags and the historical-social timeline, calling into question the importance of the temporal dimension, the study of the effects produced by hashtags inside and outside the platforms (e.g., Faltesek, 2015; Dobrin, 2020). Attention to the socio-historical and political contexts is also necessary to understand the different emotional gradients that gather around those hashtags that arise after terrorist attacks, which gain traction in different measures, depending on their ideational content (Harju, 2019). Scholars also wonder how to interpret the presence of hashtags for which the meaning does not change over time (classified as semantic invariants) and the presence of hashtags that instead undergo semantic transformation processes (e.g., Bruns and Burgess, 2015). Finally, an age-old question opens up, namely that of the relationship of influence between two contexts, represented here by the two environments that make up the media ecosystem: that of digital platforms, plus legacy media that we include within the definition of the digital platform ecosystem, and that of the social structure. This controversy finds its legitimacy in the socio-relational transformation processes that the advent of digital platforms has generated. Today we need to consider platforms as the new “custodians of the Internet” (Gillespie, 2018), and we are confronted with digital platforms that are “gradually infiltrating in and converging with the (offline legacy) institutions and practices through which democratic societies are organized” (van Dijck et al., 2018, p. 24). For this reason, van Dijck et al. (2018) prefer the term “platform society” defined as “a term that emphasizes the inextricable relation between online platforms and societal structures. Platforms do not reflect the social: they produce the social structures we live in it” (van Dijck et al., 2018, p. 24).

Platforms, therefore, intervene in the manner in which social bonds are defined by including forms of connection that mix social norms with the sociotechnical norms typical of online environments (van Dijck, 2013). Through this, they create a symbolic field and digital cultural practices that delimit specific ways of relating—often distinct from the old model of staying offline—which preside over new processes that signify being together (Boccia Artieri and Farci, 2020). It is within this socio-technical environment that the participatory practices of connected audiences and the development of social network sites, which have introduced additional elements of complexity to the transformation process underway in the last 10 years, need to be applied. Connective media (van Dijck, 2013) have become an almost uninterrupted presence in daily routines: they absorb a significant part of identity processes and social relationships, and they give life to a common heritage of cultural and symbolic practices, rules, and behavioral practices that contribute to settling “an accepted version of reality” and intersubjectively sharing it within the same communicative environment (Boccia Artieri et al., 2017).

Online platforms now preside over the socio-technical system within which all actors move themselves, carrying out a new intermediation function, which structures the information flow through logic—unnoticed at an experiential level and not transparent to all stakeholders—and the visualization algorithms on user timelines (Gillespie, 2014; Bucher, 2017). Networked publics (boyd, 2010) usually operate in a mixture of public and private spaces, within which the exchange of information (commenting, sharing, and production of content, such as memes) finds space within the flow of uninterrupted and non-segmentable communication. What we are witnessing is, on the one hand, the rise of the platform as the dominant infrastructure and economic and socialization model of the web, and on the other hand, the convergence with social media, as platforms, in building an increasingly integrated ecosystem.

At this point it appears clear that there is a need to determine the relationship of influence on the two systems, which are affected by an irreversible integration process, assuming the hashtags and social effects produced by them and through them over time as the main node. Thus, the fact that the meaning of the hashtag changes over time is evident in the studies devoted to it. More complicated, however, is identifying a model to analyse this change that takes into account the interconnection between social structures. We are in the presence of a dualism that fluctuates by polarizing two methodological dimensions, individualism and holism, in the analysis of hashtags intended as big (textual) data. To arrive at the formalization of a model capable of observing this change in the meaning of the hashtag, we need to:

a) analyse studies in which this dualism is traced;
b) analyse the time and agency dimensions of the Twitter hashtag;
c) analyse cultural morphogenesis;
d) clarify the points of contact between the two approaches; and
e) develop of model to interpret the changeable meaning of hashtags.

## 2. Previous studies on hashtags

This section reviews studies related to hashtags and Twitter, in which the platform assumes the identity of a privileged place within which hashtags were first introduced and subsequently studied (Section 2.1.). The section also explores how the temporal dimension and the agency of hashtags on Twitter are explored (Section 2.2.).

### 2.1. Twitter hashtags and their meaning

Over the past 15 years, the hashtag has become a symbol and tool of connection, which, thanks to the changes in digital technologies, has become increasingly widespread within the media ecosystem. It has been the subject of numerous studies in recent years, thereby opening up different lines of interpretation. These explanations evidently originate from the concept of folksonomies (Vander Wal, 2005, 2007) intended as the possibility for users to create classifications from the bottom-up and elaborated by groups in a freer and less official manner than the institutionalized taxonomies of the scientific community. Hashtags first appeared on Twitter in 2007, and they are defined as bottom-up user-proposed tagging conventions that embody user participation in the process of hashtag innovation because they pertain to information organization tasks (Chang, 2010). Subsequently, they have been incorporated into other social media and have aroused the interest of many scholars who have developed further insights, such as hashtags as linguistic meta-functions (Zappavigna, 2015). In this context, hashtags are viewed as being able to construe a range of complex meanings in social media texts, linking them to the possibility that they offer the opportunity to mark experiential topics, develop interpersonal relationships, and organize text. There also exists the question of “hashtag publics” (Rambukkana, 2015) that interprets them as interconnected nodes within the users' discussions which generate discursive assemblages by feeding a public discourse around a theme/event. Further, there are those researchers who stress that the proximity of hashtags to speech acts (Benovitz, 2010; Caleffi, 2015; La Rocca and Rinaldi, 2020) or defines them as narrative topos (Said and Silbey, 2018). The common theme of all these studies is that users develop actions in and through hashtags. One consequence of this user action is the change in the meaning of hashtags, which, starting from the literal meaning of its label attached to an event/theme, develops further meanings based on what users insert in their posts, such as text, images, or videos. Hashtags have potential, and their agency lies in having the power and resources to realize/manifest their potential through user action. At the same time, the collective agency allows users to co-create the possible hidden meanings of the hashtag.

In hashtags, therefore, something similar happens with respect to utterances; that is, the need to consider non-linguistic elements as determinants in the attribution of meaning matures with respect to the phenomenon of semantic underdetermination (Cruciani, 2011), whereby linguistic information alone is not considered sufficient to determine a single set of truth conditions for an utterance (Searle, 1969, 1995). Thus, when it is not possible to select a single interpretation, a speaker—in linguistics—or a user—in social media—creates through their action the interpretation that satisfies them most. From this perspective, a speaker's choice of preferred interpretation is considered a legitimate means of accounting for the attribution of meaning, as the extra-linguistic context and semantic conventions associated with the linguistic form are able to set the necessary meaning. However, the sufficient conditions for determining a single meaning are not present (Cruciani, 2011). The sufficient conditions are set by the speaker's/user's interest in the situation. Hence, the need to consider user action across time and social structures.

All these factors/conditions reconfigure the meaning of a hashtag over time, and some authors have dealt with this phenomenon. Concerning the changeability of meanings, we first find the work of Colleoni (2013), who describes these meanings as empty signifiers that invite the ideological identification of a polysemic orientation. Subsequently, Papacharissi (2016) perfected this definition by considering them as signifiers that are open to definition, redefinition, and re-appropriation, and La Rocca (2020a) defines this ability of hashtags as their “possible selves”. In addition, they signify the emotional component that users attach to events, affections, or networked publics (boyd, 2010) that express participation through sentiment expression (Papacharissi, 2016). Thus, as Bernard (2019) summarizes, the hashtag is a lingua franca that, starting from the function of a thematic aggregator, develops a network of collateral meanings (La Rocca, 2020b). However, as Bruns and Burgess (2015) point out, this occurs for topical hashtags but does not occur for non-topical hashtags, and in underlining this difference, scholars highlight the role of time, user action, and the characteristics of the linked event.

Nowadays, the prevalent idea is that of “abandoning a prehistoric view” of the hashtag that regards it as a thematic aggregator and instead viewing the “now-ubiquitous hashtag” as “an iconic symbol of Twitter” that is a “powerful part of the world's cultural, social and political vocabulary” (Burgess and Baym, 2020, p. 61–62). In fact, these days, when an event, whether it is a live concert (Danielsen and Kjus, 2019) or a football match (Adedoyin-Olowe et al., 2014), is aired through the mainstream media, it is accompanied by a hashtag which is attached to the event. This helps to create a sense of community, guarantee the development of an *ad hoc* audience, and generate media hype around the event. The same process gets repeated for political (Davis, 2013), commercial (Abidin, 2016), or public health campaigns (Laestadius and Wahl, 2017) and for forms of protests or demonstrations (Gureeva and Samorodova, 2021). Depending on what is contained in its graphic form (# + label), it becomes a connection instrument for collective responses (Abramova et al., 2022), expressions of solidarity and support (Mottahedeh, 2015), and strategies of inclusion oriented toward creating a joint sense of community that is formed around it (Golbeck et al., 2017)—within which emotions are anchored (Döveling and Sommer, 2012; Döveling et al., 2018; Harju and Huhtamäki, 2021).

These street demonstrations or mainstream media events get commented on within the digital spaces, and vice versa, producing effects in both ecosystems. Thus, the hashtag surpasses the structure of techno-social platforms and interacts with the social structure; it becomes a bridge—a link between the internal and external dimensions of the platforms. This social effect produced by hashtags was also identified by Clark (2014) in a study on #NotBuyingIt, a hashtag created “to fight misrepresentations of gender in the media *via* Twitter” (p. 1108), specifically to denounce gender stereotypes in Super Bowl commercials. #NotBuyingIt was used, during the 2014 Super Bowl, more than fifteen thousand times to report sexist advertisements, and the messages generated reached more than 2.4 million people; as a result of its sharing, companies have been forced to acknowledge feminist criticism. Meanwhile, this hashtag feminism has collected “a multiplicity of voices that demand recognition of differences across intersections of gender, sexuality, race, and class” (Clark, 2014, p. 1109), highlighting the need to create more effective coalitions between them.

From this brief introduction, some issues that are connected seem to emerge: namely, (i) how to interpret the action of users/citizens who use hashtags on and off digital platforms and help to redefine their meaning, and (ii) how to formalize the movement that a hashtag accomplishes from the time that it is introduced to the time that it reconfigures its new meaning.

Regarding the first point, in the studies that deal with social media, especially Twitter, the actions of users have been analyzed using the concept of affordances (Rathnayake and Suthers, 2018). These refer to environmental properties, which activate or offer potential action by an agent; it is a concept born into the ecologic theory of perception (Gibson, 1979). Rathnayake and Suthers (2018) extensively analyzed affordances in digital environments—specifically Twitter—to argue that hashtags are affordances for “momentary connectedness”. According to the authors, Twitter hashtags are affordances for two reasons: (1) The platform allows for the creation of hashtags, and (2) different action types emerge through them. La Rocca and Boccia Artieri (2022) add a third reason: (3) The ability of hashtags to change their original meaning through retweets and quoting, and also their circulation outside the digital environment. This ability to change meaning (morphogenesis) or not (morphostasis) expresses the “variability” of the affordance (Evans et al., 2017). This is because “affordances are a relational construct that sit in between—but do not determine—objects and outcomes” (Evans et al., 2017, p. 41). Regarding the second point, the studies on hashtag activism have noted the changeability of the meaning of hashtags, but they have not produced an operational model capable of observing this change over time.

In the current scenario, it is necessary to take into consideration that we are confronted with the collapse of contexts due to the fusion of digital platforms and social structures; additionally, we are dealing with a user action that develops collective agency over time, which may be able to generate a transformation (morphogenesis), both in relation to the meaning of the hashtag and in terms of social consequences, or not produce any effect (morphostasis). Therefore, the aim of this study is to examine these phenomena and identify new interpretations. In order to achieve this objective, we propose the following: (i) a review of the studies that deal with time and agency in Twitter hashtags and (ii) an assessment of the points of contact between what emerged in these studies and what has been proposed by the sociological theories of social change. We are attempting to graft and add value to what has previously traced within the theories of social change in hashtag studies.

### 2.2. Time and agency in Twitter hashtags

Hashtag analytics show that time and agency are important aspects to consider in order to outline a new vision of hashtags and their role. Agency and hashtags' temporal dimensions are linked to the characteristics of the social media platform. On Twitter, features are represented by mentions (@), quotations, retweets (RT), and uploads inside the tweets (links, photos, images, and emojis). @mention or @reply have become a key feature of Twitter as a tool for conversations and socializing, and their usage enables the creation and maintenance of a network between users. The recirculation of messages creates visibility through hashtag use that we need to understand. Therefore, working on these platforms and analyzing public spaces open to peer discussion has become fundamental for monitoring trends that revolve around issues of public interest. As Bruns and Stieglitz (2012) explain, many retweets occur when natural disasters or crisis events occur (Bruns and Burgess, 2015), and they fall largely into a category best described as “breaking news” or “rapid information dissemination”. A large number of retweets can be attributed to a specific conversational practice on Twitter: gatewatching (Bruns, 2005). This is an activity performed by citizens in real time to disseminate, share, and comment on news that originates from media outlets on social media platforms during acute events that generate an *ad hoc* community.

Thus, certain events produce a high percentage of retweets and certain other events/hashtags do not generate an online conversation flow (Bruns and Stieglitz, 2012). Depending on the theme, Twitter users adopt different conversational and relational models. It can be argued that “these distinctly divergent and stable patterns in user activities for different hashtag use cases indicate various conceptualisations by users of the hashtag community that they are either seeking to address or participate in” (Bruns and Burgess, 2015, p. 21). It is alongside these considerations that the hashtag can begin to take on new traits composed of symbolic elements and structural constraints: the former represented by their reference to meaning and the latter by digital platforms and network dynamics. The *ad hoc* audiences generated through the conscious use of the relational social form hashtag assume the traits of the relational public theorized by Starr (2021, p. 2), who argues that “the public depends not only on what individuals think but on how they associate and are linked with one another and develop mutual awareness and recognition”. In his study, Starr enhanced the concepts proposed by Tarde (1969), such as the intersubjectivity of the public, then activated by journalists in two ways: “first, by awakening connections among their readers, who were simultaneously learning about the same stories, and second, by triggering in-person interaction, ‘the conversations of the day”' (Starr, 2021, p. 2). These definitions fit perfectly into the dynamics created by the users on Twitter, for example, the use of hashtags to monitor issues of public interest and not to simply disperse information, or the flock of retweets that breaking news can generate.

Bruns and Stieglitz (2012) argue that hashtags linked to a crisis, event or a television program (topical hashtags) are much more likely to induce information sharing that engages and disseminates other information conveyed by URLs, photographs, and continuous retweets compared to what occurs in the case of hashtags with generic content and meaning (i.e., non-topical hashtags such as #mom, #party). Conscious hashtag and feature use is affirmed on Twitter to the point of developing the capacity for collective action on specific topics/events (Sharma, 2012; see also Bonilla and Rosa, 2015; Jackson and Foucault Welles, 2015; Rambukkana, 2015; Lindgren, 2019; Jackson et al., 2020; Olmedo Neri, 2021). Based on this, we can distinguish between topical and non-topical hashtags (Bruns and Burgess, 2015) and expand on the differences between them:

- Topical hashtags are related to themes and/or unforeseen events that can generate *ad hoc* audiences that use them consciously to create influence inside and outside the digital ecosystem by expressing awareness of themselves as activists and the strength of their actions. This enables a reconfiguring of the meaning of the event for which they were generated and their literal significance. This change is observable during a given period.
- Non-topical hashtags have a generic meaning not directly related to topics and/or events on the agenda or in the public attention. They do not generate an *ad hoc* audience but reference the denotative meaning in the historical-social context.

Topical hashtags can develop a redefining, reflective, and intersubjective action that acts on themselves and the external context of social media platforms. Therefore, this dimension can be expressed as follows:

- Reconfiguration of the symbolic meaning of the object/theme/event to which the hashtag is linked. This action is due to the conversational flow that users activate on the platform and which, similar to brainstorming, leads people to think about the event that generated them, showing their connective and relational capacity.
- Reconfiguration of its narrative meaning. This action occurs through the process of metamorphosis; that is, users' action produces the literal meaning of the hashtag over time by unfolding the temporal dimension.

Yang (2016) analyzed the narrative agency in the case of #BlackLivesMatter and defined it as being generated by online activism, thereby defining it as the outcome of an action by individuals who link a social or political claim under a common word, phrase, or hashtag. The temporal unfolding of these messages, mutually connected in networked spaces, provides the shape and strength of a narrative agency. One can consider the form of discursive protests on social media under the use of #BlackLivesMatter, a hashtag generated in response to the acquittal of George Zimmerman in July 2013 in the fatal shooting of African-American teen Trayvon Martin, which produced a protest movement both on the streets and social media networks. The same mechanism operated for #Ferguson, which appeared in response to the police shooting of Michael Brown on 9 August 2014 in Ferguson, Missouri. Within the first week of Brown's death, millions of posts with the hashtag #Ferguson appeared on Twitter alone (Bonilla and Rosa, 2015). From these uses, we can perceive the “power of digital activism in shaping public discourse” (Yang, 2016, p. 13). To explain the characteristics of the narrative agency, Yang used existing theories in literary studies and psychology, crossing the narrative and processual dimensions of stories and the subjective construction of reality by individuals through the narrative of what happens to them every day, thus emphasizing intersubjective reflexivity. In the study of social movements, these narrative and procedural characteristics have already been highlighted by other theorists. For example, Steinmetz observes that “narrative thus has a beginning, a middle and an end, and the movement toward the end is accounted for by conflicts, causal explanations and the sequence of events” (Steinmetz, 1992, p. 497). Campbell (2005) defined rhetorical agency as the capacity to act: to have the competence to speak or write in a way that will be recognized or heeded by other community members. Drawing from these studies, Yang identified the strength of the hashtag in the narrative agency explaining it “as the capacity to create stories on social media by using hashtags in a way that is collective and recognized by the public” (Yang, 2016, p. 14). This narrative agency results from a collective action—which, due to its characteristics, can be defined as a corporate agency—conducted by a relational audience that develops over time and through human interaction. The consequences of action and interaction affect changes in the object and subject over time. This is the case analyzed by Jackson and Foucault Welles (2015), who show how the action of users is able to generate counter-narratives. The hashtag they analyzed is #myNYPD launched by New York City Police Department in April of 2014 as part of a public relations campaign and with the aim of inviting citizens to share photographs of officers captioned with the hashtag #myNYPD. “What started as a public relations campaign quickly turned into an online protest as thousands of citizens appropriated the #myNYPD hashtag to highlight instances of police brutality, abuse, and racial profiling” (Jackson and Foucault Welles, 2015, p. 1). Thus, what began as a public relations campaign morphed—through user action—into a form of media activism. The two scholars rightly define this appropriation and overturning by users of the original meaning of the hashtag #myNYPD as a hijacking that generates counter-publications and counter-narratives. As Blevins et al. (2019) have already pointed out, social media offer significant opportunities to disrupt and redirect dominant and oppressive narratives through conceptual and ideological hashtags. Using these hashtags, affective publics (Papacharissi, 2016) relate “the meaning of these events to their own lives and framed these occurrences as relatable to a broader array of personal experiences and incidents” (Blevins et al., 2019, p. 2). This has an impact in terms of hashtag usage patterns and spikes and broadening the meaning of the hashtags themselves.

Narrative agency can also be found in Dobrin's (2020) study on #MeToo that emerged in response to the sexual harassment scandal involving the high-profile Hollywood producer Harvey Weinstein. The study examined the cultural importance of digital activism and the hashtag, emphasizing its symbolic role in the emerging myth around the movement through its narrative use by producers (Dobrin, 2020). Examining the hashtag's representation and mechanics under the cultural dimension allows us to read it as a cultural object that perpetuates the phenomenon's political agenda in the digital public sphere and bridges personal and collective experiences as the hashtag transforms and reconfigures the identity of the movement outside the digital environment. Dobrin's approach is culturalist, and the analysis highlights how the collective use of the hashtag has, over time, contributed toward changing the identity of the movement. Another effort in the analysis of narrative agency performed by Twitter hashtags is the one described by Dawson (2020), again for #MeToo. The scholar focuses on the role that narrative plays in the emergence of cultural movements and identifies a new narrative phenomenon created by the technological affordances of Twitter, which he defines as “emergent storytelling”. To explain it, he highlights the dynamic forces that circulate in and through Twitter, “he interplay of narrative cognition with stochastic viral activity and the invisible design of social media algorithms; and the varying rhetorical purposes that narrative is put to in public discourse about viral movements” (p. 9).

This review of studies helps us to understand a few important aspects:

- Despite being just a word, a hashtag has much more power than the word representing it.
- Its physiognomy changes through human action and interaction, and
- Time, understood as the use of hashtags over time, is an essential element to understand its origin, development, change, and impact on social structures.

These effects, which are derived from hashtag use, are possible because of affordances (Gibson, 1979; Hutchby, 2001) of the platforms, the dynamics of digital environments, and many-to-many communication modes. However, according to the indications emerging out of hashtag activism, we additionally need to start considering the effects produced outside the digital platforms and the manner in which what is external and internal to the platforms have merged into the dynamics produced by hashtags. According to a review of studies, these dimensions are relevant for topical hashtags. The research dimension develops alongside this content–contextual dimension where scholars attribute meaning to these elements and the semantic and semiotic reconfiguration of the hashtag. As Burgess and Baym (2020) explained, the hashtag has become a ubiquitous symbol that enters language, social, political, and cultural contexts; one must deal with multiple dimensions that must, in some way, make their link explicit. For example, Faltesek (2015) argued that the temporal question of public hashtags exists only because of the attention and recirculation attributed by the event that generates them. Faltesek recommends paying “particular attention to the temporality of their circulation as part of an unpredictable flow of messages that is both tightly controlled and beyond control at the same time. This practice of reading, thinking and engaging must take place in the real-time of the researcher since the traces left by hashtag are a poor substitute of the phenomena itself” (Faltesek, 2015, p. 84).

Faltesek linked time to the collection and interpretation of hashtags and to the historical-social timeline and events that generated them. He released them from their strict links to digital and research functions on social platforms and instead anchored them to a cultural and social dimension. He drew a distinction between (i) kairos, which is a “rhetorical moment or situation”, a moment of an indefinite period in which “something” special happens and (ii) chronos, which refers to chronological and sequential time. On Twitter, the first—kairos—cannot be determined solely at an individual level because it is represented by read or unread tweets or by the significant contexts that the elements of polysemic digital communication allow by linking to hashtags. Instead, they must be sought in the temporal (and social) context within which networks were generated from the hashtags and in which they are located and operate by activating an *ad hoc* public that follows specific hashtags, tweets, retweets, and uses them in combination with other hashtags. The approach developed here goes beyond Faltesek's considerations and stresses this temporal dimension because it allows us to identify the ability of agents to modify the meaning of a hashtag (morphogenesis) or to leave it unchanged (morphostasis).

Vicari (2017) studied the flow of tweets posted over two sample periods, namely 14 April−13 June 2013 and 24 February−23 April 2015, linked to the declaration by actress Angelina Jolie that she would undergo preventive surgery as a carrier of the #BRCA gene mutation. Vicari highlighted the “need to look at the life story of issue-based Twitter streams to fully understand the changing role of social media platforms in enhancing old and new power structures underlying discursive practices” (p. 2).

Furthermore, Bruns and Stieglitz (2013) highlighted the importance of working with temporal metrics in the analysis of tweets which allow a breakdown of the total dataset by time. Their strategy involves considering the timeframe of the event analyzed to identify the time unit of interest; therefore, “for hashtags relating to short-term events (from live sports to television shows), minute-by-minute analysis might be appropriate; for longer-term activities (such as election campaigns or unfolding crises), day-by-day timeframes may make more sense; for long-term phenomena from brand communication to military conflicts even a month-by-month analysis may generate useful results” (p. 99). This approach to temporal metrics seems to be related more to chronos than kairos and allows the analysis of the change in the meaning of the hashtag to remain accessible over time.

Nonetheless, Faltesek, Vicari, and Bruns and Stieglitz showed the importance of the temporal dimension: The first by linking it to the interpretative processes of the historical social context; the second to the dimension of analysis of the tweets, which must develop diachronically; and the third by linking it to a web-scraping strategy and the calculation of metrics. In a recent article aimed at bringing out the nascent traits of research using hashtags, La Rocca and Boccia Artieri (2022), working on Vasterman's (2004, 2005) theories about media hype and Pang's (2013) on social media hype, conclude that “The introduction of these elements—of time or period, of storm or engagement, of interdependence or lack of relationship between media and social media hype—builds a framework through which to interpret of the research results” (La Rocca and Boccia Artieri, 2022, p. 9). They suggest considering these two elements, because they identify the importance of the hashtag and what it links to on and off the platforms.

Previous authors' studies provide the opportunity to understand how crucial it is to observe over time—a time that we define as the “relational time” to clarify that it is the time during which the agency occurs—the construction of a relational frame of reference, from the intersection of different planes along which the collective agency manifests itself in social structures and its effects of changing the hashtag's meanings.

Therefore, hashtag analysis cannot ignore contextualization for sporting events, crises, natural and environmental disasters (topical hashtags), or generic hashtags, such as #mom, #work, and #food (non-topical hashtags), as they always carry an interpretative meaning and dimension continually linked to the social context. The difference observed between topical and non-topical hashtags is the time frame that researchers give themselves as a reference point for tracking its evolution. In topical hashtags, meaning changes occur within a short period and are observable; in non-topical hashtags, meaning changes take place over a long time and requires analysis of how the concept to which the hashtag refers is understood in different socio-cultural systems, specifically in terms of temporality and spatiality. Regarding spatiality, consider #mom or #food and their implication in a European context or the evolution of a signifier in the same context but different centuries. Therefore, before starting the interpretative process—a posteriori—of a hashtag, it is necessary to specify the meaning of the “word” in that culture. Second, by reconstructing the meaning connected to the hashtag, we can indicate how much they differ from the current use, if they align with it, or how much they contribute toward redesigning its morphology. This implies that in analyzing hashtags, researchers acknowledge they are complex social entities (La Rocca and Boccia Artieri, 2022) alongside two dimensions: hashtag as a “sense-making practice” and as a “propagation-effect practice”. Therefore, there is a conceptual dimension/category that descends upon the hashtag a priori and is represented by its literal meaning, and this is followed by reconstruction, a posteriori, of the meaning and effects co-created by the users' actions. Considering a change in meaning over time requires looking at the hashtag itself differently. This process would have practical repercussions on research design, requiring a specific model to interpret the changes.

## 3. Emergence of the hashtag as a relational social form

The examination of studies related to issues concerning the actions of hashtag users reveals the consequent possibility that their meaning could be reconfigured. It also highlights the fact that this effect needs to be observed over time and that it manifests itself inside and outside the digital environment, broadening the way we view the hashtag. For this reason, we propose a new interpretation which originates from the idea that hashtags can be considered as a relational social form. Evidently, the change that has taken place within the media ecosystem plays a fundamental role in this proposal, because today, as already pointed out at the beginning of this article, we are confronted with digital platforms that are “gradually infiltrating in and converging with the (offline legacy) institutions and practices through which democratic societies are organized” (van Dijck et al., 2018, p. 24).

It is from this convergence that the need to identify new dimensions of reference for the hashtag emerges. It is about considering how the meaning of a hashtag changes according to interconnection logic. This interconnection is systemic, because it not only concerns social structures, but it also concerns the possibilities of action by the users/citizens and the hashtag itself. These are the motivations that lead us to look to sociological theories dealing with social change to find theoretical frameworks capable of focusing on this process of changing the meaning of the hashtag. The concept of change seems to make sense only when related to something, that is, when there is a change with reference to an already given or existent order. Rocher (1973) essentially defines change as a collective phenomenon, which concerns the collectivity or a part of it, and which consequently involves the conditions, ways of life, and of thinking of an individual. This consideration is applicable also to hashtags and to the dynamics underlying the change of meaning both in topical and non-topical hashtags. Social change needs factors and conditions to take place (Rocher, 2004, p. 379) claims that “the historical actions of human beings take place through a great number of conditions, factors, constraints that more or less favor the attainment of human goals. Some of these factors are attached to the individual and human action, while others are external to the individual”. A factor of change is a strong determinant of social change itself. This means that a factor can be considered an element of a given situation, that for the simple fact of existing or for the action it performs, provokes, or produces a change (Rocher, 1973). Instead, conditions are the elements which favor or inhibit, feed or slow down, encourage or delay the influence of one or more factors of change. There are, therefore, structural or material and cultural factors; the former include demographic, technological, and economic infrastructural factors, and the latter cultural and ideological ones. In the analysis we carry out here, the structural factors are identified as products of the platforms and their relational and market logics. The cultural ones are instead to be found in the network dynamics of the users, which show their effects within and outside the digital ecosystem.

Here, we assume hashtags are a subject of observation for themselves, their function, and their social effects, linking them to a temporal dimension and the results produced by a collective agency. We then interpret hashtags as a manifestation of a new form of social relationship because they move within two environments—the digital platform ecosystem and other social structures—interacting with the structural dimensions and connections (*re-ligo*) of both, and with the different references of meanings that are generated progressively (*re-fero*), thereby creating a reciprocal action that generates a third party, namely hashtags, as a relational social form.

The hashtag as a relational social form is an “emerging property” generated by the interaction of users/citizens—inside and outside digital environments—behaving like “corporate agents” (Archer, 1995) who organize their actions in a formal or informal way (Karlsson, 2020) to reach their goals. The result of their actions is defined as a “corporate agency” and gradually manifests itself as a structural transformation or morphogenesis. This perspective allows us to highlight that the hashtag is determined by a strong semiotic charge for the word that forms it, represented by the power of sharing. This power is determined by using hashtags and the gratification received while generating and regenerating them. The regeneration of the meaning of hashtags is determined by the circulation process that places the ownership of the connotation into the hands of those who share them, thus developing a network of collateral meanings and actions which produce a morphogenesis.

Here, we have begun to outline the elements that allow us to consider the hashtag as a relational social form, which means that it is possible to think that a grafting of the relational approach could be practiced on it. Accordingly, it is possible to read hashtags through lenses that are certainly not new but can instead be considered vintage.

In the first section of this work, the reader was introduced to the notions of time and agency in Twitter and to how they produce effects inside and outside the medial ecosystem. In other words, the reader understands how they produce social change. We now wish to outline the aspects of cultural morphogenesis (Section 3.1.). We are looking for a theoretical matrix capable of explaining the change inside the hashtag. One that is linked to the hashtag's signification processes and to the external changes it produces in the social system.

We define this application of social change theories to hashtag studies as vintage glasses because they were produced at least 20 years before the present moment, and they are “grafted” because we are joining two theoretical perspectives that belong to different fields. The definition of hashtags as a relational social form—outlined above—is based on the relational approach (Archer, 1996; Donati, 2011), on the social relationship (Donati, 2006), and on the decline of the morphogenetic sequence to social forms (Donati, 2006). Furthermore, this grafting that we are practicing provides us with a key to understanding the gradual change in the meaning of the hashtag, allowing us to insert it within the cultural morphogenesis.

### 3.1. Cultural morphogenesis

The concept of change makes sense only when it is related to something—therefore, an *a posteriori* compared with an *a priori*—that is, when there is a change concerning an already given or existent order. The same consideration can be made for the meaning of the signifier and the signified in hashtags: (1) the signifier's change is assessed by considering the transformation in the content conveyed and collected by the hashtag, (2) and the signified's change is the impact of the collective action on the hashtag's meanings and its effect on the theme/event/movement from which it originates. The concept of change makes it necessary to refer to a context or system and to the already existent dynamics, which can be internal, external or interrelated.

In essence, the same “problem” that arises in sociological theories arises within hashtag study and analyses, i.e., whether it is the agency that produces a change in the social system or whether social structures act on individuals.

Archer emphasized the dualistic relationship between structure and action stigmatizing the previous approaches as unidimensional theorisations characterized by a conflation, a fusion upwards, downwards or toward the center of the issue i.e., individual versus society. It is an old and a thorny dilemma, interpretable in an almost Hamletic sense, that is, whether the change is to be attributed to structures, to the individual or the action. In the untangling of this dilemma, traditional “conflationists” have opted for one or the other option from time to time, putting aside the idea of the possibility of a sociological dualism, in which the different aspects refer to different social reality elements. The interrelations and interconnections between these properties and capabilities are the core of non-conflative theories whose characteristic is that these aspects need to be related rather than led back and absorbed into each other. In this opening, non-conflative theories can explain hashtags' change. The essential aspect of Archer (1995) analytical dualism, that is, the morphogenetic-morphostatic approach, offers the basis for observing the morphogenesis of hashtags. Archer proposed the morphogenetic-morphostatic approach based on the understanding that society is similar only to itself, and the fundamental task is to understand how social forms derive from human action exactly as social beings derive from social forms (Archer, 1995, p. 255).

Incorporated into the context of transformation, change and mutation, this approach of morphogenesis and morphostasis makes an analytic distinction between action and structure, unable to claim that human beings create the structure but affirming that they reproduce and transform it. Social structure is always a given when referring to *intentional human action*; the social practice has a restructuring effect, which can be the starting point of a new cycle of action and change.

The essential aspect of this approach, which considers cultural and communicative change, is that it enhances analytical dualism as the criterion used to analyse structure and action, and it recognizes its existence for the analysis of cultural systems and socio-cultural interactions. The analysis of cultural change includes four equivalent propositions based on the identification of properties belonging only to cultural systems and that do not melt down into the characteristics typical of socio-cultural interaction. Hence, there is a morphogenetic cycle of cultural conditioning, cultural interaction and cultural elaboration in which cycles are considered constant and the final product of the elaboration of cultural system contributes to the creation of logical and necessary internal relations between the components of the cultural system itself, creating a new cycle of cultural change. Archer's approach also concerns cultural systems and socio-cultural interactions, offering the basis for observing the morphogenesis of hashtags as *emergent properties* determined by the interaction of a group of individuals belonging to a certain social structure that already contains its own cultural and value-driven orientation. Archer (2007, p. 39) claimed “constituted as human beings are, the world being the way it is, the interaction between the two is a matter of necessity”.

To explore this interaction, we must determine the conceivable properties capable of producing any kind of causal effect attributable to the social systems considered together with individuals and which, in any case, are an influence independent from them. When proposing this approach, Archer analyses these issues as they have been tackled by social realists and institutionalists. We accept the morphogenetic approach that emergent properties are different from the evident and long-lasting patterns of social life because they are not observable characteristics of the structural domain as “institutional models”, “social organizations” and the “socio-economic classes” (Archer, 1995). However, they are not observable characteristics of the cultural domain because of their heterogeneity, represented by a mix of aggregations, people and positions. Furthermore, this heterogeneity is inevitable because it emerges from the observation of events that are collected and categorized, and it incorporates a series of contingent regularities based on a series of undifferentiated sources. The unity possessed by the event categories and structures is given by the observer who categorizes them as objects of observation. Hence, considering the internal and necessary relations between the elements as constitutive of an emergent property means separating them from external and contingent relations.

There are numerous emergent properties in society, namely structural, cultural and action-related. Each is irreducible to the others, being relatively autonomous and long-lasting. Another fundamental element is the one linked to the primary dependence of the emergent structural properties on material resources. This idea implies that the latter makes the emergent properties what they are and without them, the properties would not exist or possess the causal influence they are characterized by Archer (1995).

When we state that structural emergent properties cannot be reduced to individuals and are relatively long-lasting, we must distinguish between them and their unexpected consequences. What distinguishes them is that the unexpected consequences are defined as “aggregated”: even if the effects produced are important, they can be broken up and disaggregated into a sum of individual actions. Hence, emergent structural properties are not determined by additive actions but are an expression of internal and necessary relations between real collectivizes and their relations with external entities.

Culture and its emergent properties can be similarly outlined to the structure and emergent structural properties. Therefore, in analytic dualistic terms, the elements of the cultural system are pre-existent and autonomous, which identifies them as different entities from the meanings perceived and attributed by the actors in the moment. This distinction is possible because there are logical relationships between the objects that make up the cultural system while causal relationships exist between the social agents (Archer, 1995).

In the cultural system, it is easy to understand that the systematization of beliefs, symbols and artifacts has been carried out by previous thinkers; the architecture emerging from this systematization reveals a logical relationship between the parts, which can also be based on independent interaction. In contrast, the attribution of causal relations depends on agents.

Analytical dualism, therefore, consists of distinguishing and defining the cultural system (CS) and the level of socio-cultural interaction (S-C). Components in the model are ideas (CS) and human beings or persons (S-C), respectively. Thus, “the level of the Cultural System is ruled by logical relations and the socio-cultural interactive level by relations of causality rooted in human intentional agency” (Hałas, 2017, p. 124).

Culture is a human product, and it escapes the control of its creators. Therefore, the CS contains constraints in addition to new possibilities. It introduces new issues linked to relations between emergent entities, between the physical environment and between human agents. This statement has been criticized and could lead to an *aporia*; however, Archer's creation of a temporal framework determines the appropriate context to identify such emergent properties. In this way, CS properties do not depend on what happens at an S-C level because at a time defined as T_1_, the logical relations are independent of the causal ones. All CS elements have escaped from the control of their creators and can develop reciprocal logical relations independently of what people think, feel or know about them (Archer, 1995). It can also happen that in a future moment—considered as the product or passage from T_2_ to T_3_–the agents' actions will have a significant impact on the universe of the CS to be inserted in the register of the latter (T_4_). If this incorporation takes place, this new register or CS can escape again from the control of its creators who will immediately develop logical and reciprocal relations with the previous ideas (Archer, 1995).

If we insert hashtags and their changing action into Archer's analytic dynamism, the change appears as an emergent CS property expressed by the interaction of individuals and groups with the existing socio-cultural system. The property emerges from a pre-existing system of cultural values and results from a concatenation of events observed in their regularity, thereby creating a new cycle and expressing a morphogenetic capacity to react to a given system. Being connected to events, it is not a theory, but a phenomenon destined to endure the time required to accomplish the tasks it was created for and capable of producing a new bifurcation that can result in a morphostasis or morphogenesis.

## 4. Touchable points

A question which arises is whether it is possible to graft the relational approach onto hashtag studies in order to create a new manner of reading hashtags. Before proceeding with a graft, it is necessary to study the conditions of engraftment and one of them is represented by the affinity or, in this case, the proximity of the themes and approaches.

Moving from Archer's morphogenetic-morphostatic model (M/M), we have three stages: (1) at T_1_, there is an existing structure, which is the result of past interactions between different agents; (2) at the period T_2_-T_3_, there is an interaction between agents connected to the structure in question; and (3) finally, the outcome at T_4_ can either be that the structure is reproduced (morphostasis) or transformed (morphogenesis). The stages can be represented as cultural conditioning → social-cultural interaction → cultural elaboration, thus stressing the interplay between structure and agency. There are two different types of agents in this model, “primary agents, the agency that results in structural reproduction or morphostasis, and corporate agents, the agency that results in structural transformation or morphogenesis” (Karlsson, 2020, p. 46).

Hence, we believe the same morphogenetic-morphostatic process can characterize the change underlying the signifier and the signified in hashtags. To support this statement, it is necessary to explain the points of contact between morphogenetic cultures and morphogenic hashtags.

1. First, consider Archer's claims in *Culture and Agency: The Place of Culture in Social Theory* (Archer, 1996). Culture is a part of SAC (structure, agency and culture); thus, it forms one of the constitutional layers of the social order alongside structure and agency (2015, p. 155). The author contends that there is no hierarchical order between these three elements as they are constitutive of the phenomena that belong equally to the micro, meso and macro levels of social reality where networks of social relations are inherent and continuously reconstituted in time through morphogenetic cycles. Furthermore, Archer states, “if structure and culture do have relative autonomy from one another, then there is the interplay between them which it is necessary to explore theoretically” (Archer, 1996, p. 27). Connective media (van Dijck, 2013) are an uninterrupted presence in daily routines: they absorb a significant part of identity processes and social relationships and give life to a common heritage of cultural and symbolic practices, rules and behavioral practices that contribute to settling “an accepted version of reality” intersubjectively shared within the same communicative environment (Boccia Artieri et al., 2017). Online platforms now preside over socio-technical systems within which all actors move, performing a completely new intermediation function that structures the relational flow through logics that are not yet fully understood. This is confirmed by other studies (Goel et al., 2015; Benkler et al., 2018) which were used by Starr (2021, p. 15) to show the nature of relational public and underline how “mass communications” and “social networks” have converged in the analysis of contemporary media.
2. Second, according to Archer, culture, despite being autonomous and not subject to the social structure, plays an essential role in revealing the mechanisms of social change. Culture does not act alone or automatically but through the reflexive agency of actors who can articulate the principles of this morphogenic change as they are conscious of its ideational orientation (Archer, 2012, p. 33). This process recalls the mechanisms based on the formation of *ad hoc* publics created around hashtags which produce social effects. The SAC model proposes to view culture as a realm of properties and powers that are in constant interplay; in other words, a realm of cultural dynamics (Archer, 1996, p. 101) where the hashtag as a cultural product (e.g., Dobrin, 2020) finds its reason to be inserted and observed in this model. The analytic distinction in the SAC model allows us to develop a morphogenetic approach to the study of culture based on the following facts that: (1) ideas are *sui generis* real, (2) idea sharing is contingent and (3) idea interplay from the level of the CS and the level of S-C interaction leads to a new morphogenetic cycle phase called cultural elaboration (Archer and Elder-Vass, 2012; Archer, 2017, p. 163) that is useful in the development of a morphogenetic-morphostatic hashtag model (model M/M-H). We need to clarify the use of the term “idea” inside the SAC. For this, we review other authors starting with Popper (1978). Notwithstanding other views (Porpora, 2015), we are interested in Archer who conceptualizes that culture is inside the CS and it is ideational; however, it is used in various ways on the S-C level because people believe it is “a repertoire of ideas for construing the situations in which they find themselves” (Archer, 2017, p. 155). Studies on hashtags are based on sharing a set of denotations that become part of the situation's definition which is evident in the change of the signifier and the signified of a hashtag and the action performed by *ad hoc* publics that transform the sign # and its label into a socio-relational form.
3. Consider that the M/M model has been criticized and improved (Karlsson, 2020); despite the open debate in the ambit of social theory, it allows us to combine two disciplines that are working at the individual action level and on the change that is determined on a social form (i.e., the hashtag), which is capable of moving out of one environment to contaminate another. Thus, it is possible to claim that at T_1_, a hashtag is inserted on platforms and inevitably has a relationship with the social system. The meaning at time T_1_ is determined by the social context. At T_2_-T_3_, there is a relational interaction between the agents connected within the definition of *ad hoc* publics. At T_4_, its meaning is reproduced (morphostasis) or transformed (morphogenesis). Topical hashtags can fall into the morphogenetic approach because they change over time. Meanwhile, hashtags that are not topical can undergo a morphostatic process due to their not changing the meaning in a specific period. However, in the morphogenesis-morphostasis assessment, we must specify the observation period. Furthermore, in this case, in the morphogenesis-morphostasis assessment, we need to specify the time span of the observation. T2–T3 can be observed under the lens of “transitional temporality [that] identifies the capacity for agents to alter the rate of change between events and even to prevent or bring about events by speeding up or slowing down transitions” (Hirschman, 2021, p. 49). This concept remains valid whether the morphogenesis takes place or the morphostasis occurs because as Hirschman (2021, p. 49) explains, “(f)or scholars pursuing research under the banner of transitional temporality, the journey […] remains important even if the destination is unchanged”. This type of temporality emphasizes the agency between, rather than during, events. It is a form of temporality that acquires as the object of observation the unfolding “of some local process that was in turn set in place by some prior contingent process. In other words, transitional temporality is at least roughly compatible with eventful temporality, but it focuses on the periods between events rather than the events themselves” (2021, p. 52). Within it, the research questions seem to be the same as those asked by the scholars of hashtag studies, that is, what is the speed with which the change takes place and the hashtag is spread; in what way do the systems on which the hashtag has an impact accept or reject the instances that are vehiculated through it and when do the instances vehiculated through the hashtag determine a real change.
4. The M/M model considers the influence of the temporal variable which is fundamental in hashtag studies (Bruns and Stieglitz, 2013; Faltesek, 2015). First, reasoning on the temporal issue of hashtags makes us reflect on their internal dimension, represented by the fact that they are contained and used within the structure of proprietary platforms. Therefore, their external dimension is connected to events emerging from a socio-structural context. Hashtag time is the intersection between a digital dimension (internal and soft) and a socio-structural (external and hard) one. These two dimensions determine the peculiarity of the nature of time that takes shape with and through space and experience. However, hashtag time is the time of oblivion and non-oblivion because a hashtag survives if it circulates, that is, if it is used; otherwise, it goes unheeded and becomes a frill inserted in a short text. The time of a hashtag reveals its importance: if it succeeds in passing from T_1_ to T_2_ and T_3_, in other words, if it finds strength in the interaction and releases the agency in the sharing, then, it is an expression of interest. If it reaches the T_2_ and T_3_ phases, the hashtag enters the morphogenetic circle through a corporate agency.
5. The centrality of the cultural dynamic problem in morphogenesis is another element that favors the inclusion of hashtag changes in this approach. As explained above (see point 2), Archer's approach considers culture as a set of properties and powers that are constantly interacting and full of cultural dynamics. At the basis of this cultural dynamism, appointing is essential because the nominal forms used in language to categorize reality appear to substantialise or even reify it (Elias, 1978, p. 112). Topical hashtags are considered *de facto* as socio-relational forms and reify reality by categorizing it. This is evident in some studies that examined hashtags as a linguistic act (Benovitz, 2010; Caleffi, 2015; Rambukkana, 2015) and as pertaining to a shared semantic field. In some studies, the hashtag has been associated with the illocutionary act of Searle's theory (Searle, 1969) and his works on the construction of reality (Searle, 1995). We can consider a hashtag as a speech act (La Rocca and Rinaldi, 2020) because the way in which we indicate “things” and the words we use determine what we know, what we maintain as an idea and what we give a representation of. However, we must take into consideration another element, namely that the hashtags that we use on Twitter intrinsically include digital characters made of bits that pass from one state to another very quickly. The core of the issue is exactly the following: in the passage from one state to another one, we digit, tweet and retweet the hashtags until they perform certain functions, but these functions are always related to the observer. It is only because the observer knows how to interpret a hashtag and what it recalls that we can claim that a hashtag contains information in itself. As Searle explains, in speech acts, there is a sense of intentionality independent from the observer (intrinsic intentionality), a sense of intentionality extrinsic to the observer (extrinsic intentionality) and then a third form of intentionality, i.e., the metamorphic one. This distinction made by Searle is without doubt relevant when one needs to extract the meanings of hashtags and their re-modulation in the various uses by social media users. Hence, we always need to distinguish between the literal use of the hashtag's intentional concepts, the literal use that describes intentional states intrinsic or independent from the observer and the literal use that describe intentional conditions so visible only to the observer. These two literal applications of intentional notions should be, in turn, distinguished from the metaphorical applications of intentional notions. Searle's clarification is fundamental in that it is impossible to have an intentional state without having several others. Indeed, the intentional state presuppose beliefs, values and wishes, and we can consider them as a web in which every intentional state works. In other words, the intentional state determines one's conditions of satisfaction only for its position and relation to all the others on the net or in social media. The entire net of intentionality works only because there is a background, i.e., a field that makes it possible for the detailed elements of the net to work adequately. Such a background does not consist of further beliefs added to the net itself but rather of a habitus, shared social practices and ways of being correlated to it in some way. For these reasons, the hashtag becomes a semantic umbrella, a polysemic connector and a collector of emotions that are always correlated but which also extend and redesign the nuances of sense and of the original meaning.

## 5. Discussion: Toward an interpretative model

If the SAC model is merely a toolkit to assist in the study of social and cultural dynamics (Hałas, 2017, p. 124), then what we are outlining here is essentially a toolkit to study and analyse hashtags. The reasons M/M-H is a toolkit are evident when one works with hashtags, in other words, when they become research subjects. To work with and on hashtags, following the indications of the M/M-H model, we must consider the *a priori* meaning of the hashtag (H#) and measure if the interaction has produced—*ex post*—a change in the signified and in the signifier, that is, in the hashtag (h#) form itself. We then indicate using H# the *a priori* meaning of the hashtag (#) and with the h# form indicating that which unfolds through the interaction.

The formalization is:

- If # at T_1_ is = a # at T_4_, then the morphostasis is H#
- If # at T_1_ is ≠ a # at T_4_, then the morphogenesis of H# in h# ⇔ T_2_-T_3humaninteraction_
- if T_1signifier+signified_ ≠ T_4signifier+signified_ ∧ H# ≠ h#

Hence, at T_1_, we have H#, which possesses a predated signifier and signified. If at T_2_-T_3_, the signifier and the signified of # change through interactions, we have a morphogenetic process indicated by h#, which allows us to evaluate how the signifier and the signified of # have changed at T_4_. If the interaction at T_2_-T_3_ does not determine a change in the signifier and the signified of #, then at T_4_, we register a morphostatic process. The temporal journey of hashtags develops along a relational temporal dimension.

**Figure 1:**
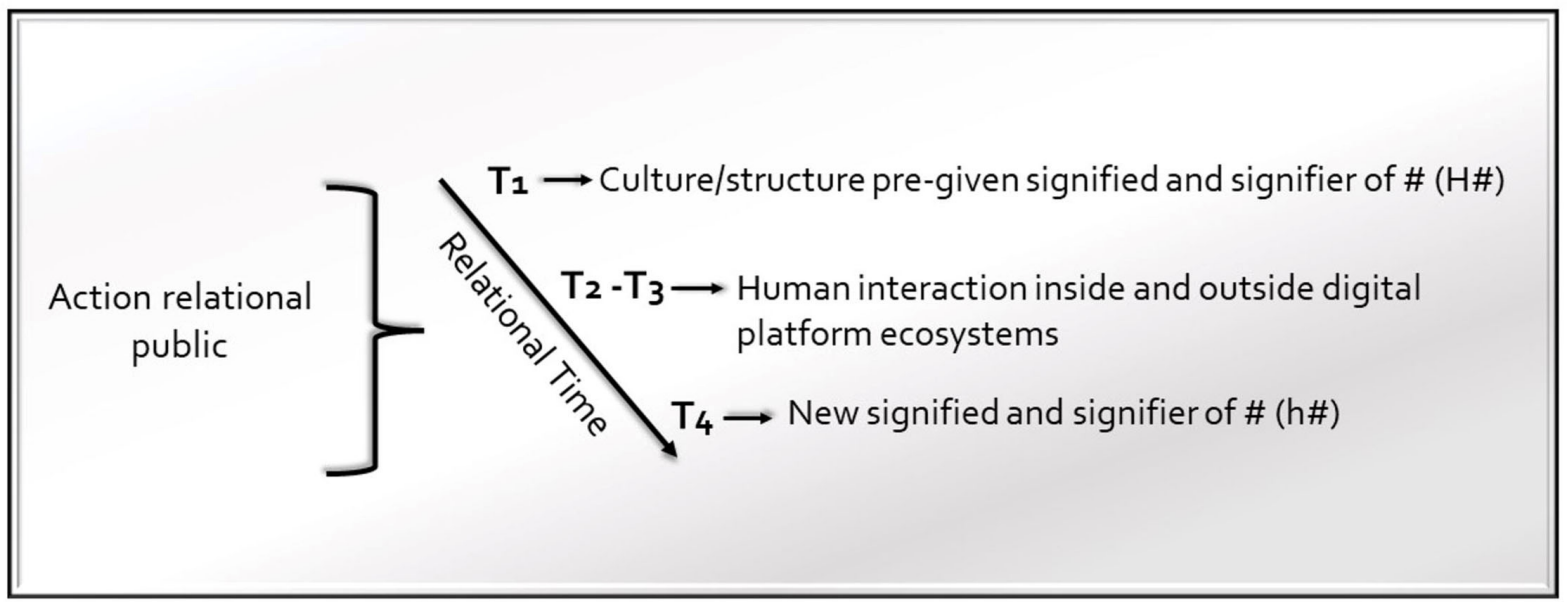
Morphogenetic-morphostatic model of hashtags (M/M-H).

Indeed, the remodeled hashtag (h#) can be considered as a reality *sui generis*, which shapes a full and embedded social relationship comprising a symbolic dimension (*re-fero*) and structural ties (*re-ligo*). This result emerges through the action of a relational public, which is an “open-ended network of actors linked through flows of communication, shared stories and civic or other collective concerns” (Starr, 2021, p. 21). The definition of hashtags eliminates their analytic dilemma because it is possible to trace in their being a socio-relational form of all the elements that allow them to embrace the morphogenetic cycle and reveal their morphogenesis. Hashtags are an expressive-communicative-social form that belongs to the dynamics and ideas of the CS, and they unfold their changes in the interaction happening within the S-C system. As Archer outlines, we recognize that a hashtag (H#) transforms inside the S-C, showing at T_4_ h# (see Figure 1). The relational public acts along a relational temporal dimension creating a transformation in the hashtag's meaning. Thus, the hashtag reports complex relationships.

Additionally, we recognize that in every new cycle, hashtags can be unlike the previous ones. This scheme moves along three fundamental acquisitions: awareness of the relational public, the relationality of hashtags and the expression of the agency. The introduction of the relational temporal dimension as the interpretative key of the temporal cycle highlights how actions always have a predated direction—they move within and along with a social structure—while changes and transformations occur when we move in that direction. These transformations can be rapid, and during the unfolding of these actions, we must consider the agency. The relational temporal dimension provides a lens to understand the agency outside the events' context and how it may help explain the timing of events. This permits us to examine changes taking place inside the hashtag by showing us how they develop through a process of interactions that reveal the intentionality of the relational action of the public connected online.

The definition provided here for remodeled hashtags contains a few concepts developed by Donati (2006, 2011) and they always move within the relational approach (Donati and Archer, 2015). Thus, it is possible to develop an approach capable of highlighting the hashtag's reality as an emergent relational phenomenon. The premise is based on the fact that a hashtag exists if at least one of its basic relations exists, that is, its relationship with the structure (external and hard) and with the digital dimension (internal and soft) which together make up the potential net where it can be created and survives. Even if hashtag studies have not framed this relation within the morphogenetic process, they recognize how these relations change. The hashtag's social dimension consists of the relations it comprises, and which are formally represented inside the M/M-H model as follows:

- T_1signifier+signified_ = ∨ ≠ T_4signifier+signified_

The social aspect found in hashtags is the result of human actions performed on platforms by users, but as human beings, they move between the agency and structure and that is what generates a morphogenetic cycle characterized by structural conditioning → social interaction → structural elaboration.

According to Donati (2006, p. 112–113), relations at various levels of this cycle are as follows:

a) Intersubjective relations, both empathic and communicative, constitute the hashtag as a social form replete with meaning as a polysemic connector and an agency toolkit.
b) Relational structures, namely the ties created by the sub-cultures and expectations of the general social system they belong to, constitute its socio-relational form.

These relations must be considered in their dual nature:

c) As a reference of sense, therefore, symbolic and intentional, we find the *re-fero* relational dimension elaborated in the intersubjectivity that occurs in the context of the sub-culture of individuals who interact by developing their relationality.
d) As mutual ties, thus, as a *re-ligo* relational dimension that forms in the reciprocal expectations of communication and expectations of other sub-social systems (e.g., mainstream media) with the associated institutions they represent (e.g., public authorities or movements).

Based on the above analysis, these dimensions are relatively autonomous and interdependent. By combining these two couples (a-b, c-d), it is possible to outline the social dimension of hashtags from which they gain their connective strength and morphogenetic capacity (see Table 1).

**Table 1:** The dimensions of a hashtag as a relational social form.

**Relations**	**c) Reference of sense (re-fero)**	**d) Reference of ties (re-ligo)**
a) Intersubjective	Empathic and intentional sense, symbol reference	Ties created through mutual expectations, relationality, communication and digital platform ecosystems
b) Structures	Sub-cultures, social expectations	The expectation of social system (e.g., public institutions, networks, mainstream system, social movements)

Reworking starting Donati's scheme (2006).

(1: a-c) A hashtag has an empathic and intentional sense addressing the symbols it is made up of as a social form replete with meanings created by its users as relational public.

(2: b-c) A hashtag is an encounter between sub-cultures that represent and actualise its meanings as a meeting/clash of different social expectations. Thus, it becomes a claim maker.

(3: a-d) A hashtag is the creation of ties through mutual symbolic and communicative interactions.

(4: b-d) A hashtag is an answer to the expectations of other institutions in society.

The hashtag-h# is a complete social relation that emerges as a phenomenon produced by the interaction between all the components it comprises in the moment in which they are specified in the cultural code typical of hashtags, that is, connecting within and outside the digital platform. Both predated signifiers and signified that undergo a process of transformation are implemented through the reflective action of the relational public.

## 6. Conclusion

As is evident from its present appearance, the meaning, use and relationship with the social context of this symbol have transformed, thus breaching the manner of its working considering its use and morphogenetic processes. Several scholars concerned with hashtag studies have raised questions that dispute a few concepts that have already been analyzed within the realm of social sciences, specifically within those theories that deal with socio-cultural change. In fact, these scholars wonder how to closely examine the agency of users of a hashtag, reconfigured its meaning through their actions, and over time (Yang, 2016) question themselves on how to interpret the relationship between hashtags and historical and social timelines. Additionally, they have raised doubts about the importance of the temporal dimension in the study of the impact of internal and external changes on the dynamics of platforms that scholars are able to launch (Faltesek, 2015; Dobrin, 2020). Furthermore, they reflect on ways to interpret the presence of hashtags that do not change their meaning over time and are classifiable as semantic invariants and the presence of hashtags that instead undergo semantic transformation processes (Bruns and Burgess, 2015).

This study is part of the debate on the nature and characteristics of the hashtag, and we have tried to define it as a relational social form affected by morphogenetic-morphostatic processes with the aim of interpreting the power of social connection and change that it produces on technological platforms and social systems. To develop this interpretative proposal, the dimensions of time and agency were presented as examined in hashtag studies based on Twitter. Subsequently, some elements of cultural morphogenesis were recalled and traced and these elements, which are the points of contact between hashtag studies and cultural morphogenesis, were discussed. Finally, an interpretative proposal of the hashtag as a relational social form was constructed.

In this study, we formalized a model to examine the hashtag as a relational social form that operates on social media platforms and within contemporary social structures. This model allows the operationalisation of a hashtag because a hashtag becomes a social entity whose characteristics can be specified within a research design. Therefore, researchers can identify *a priori* the conceptual dimensions of a hashtag and subsequently, in the data analysis phase, understand how much and under which processes the meaning of the hashtag has changed and the effect it has produced on its signifier. The model's limit is that it stops at a theoretical level, and it can also be linked to the limits of the relational approach. The limits of the relational approach postulate that culture is a human product and that it too escapes its creators to act on them. This means that the cultural system contains not only constraints but also new possibilities, and it also introduces new problems related to the relationships between emerging entities, between these and the physical environment and between these and human agents. However, Archer (1996) already inserts a temporal scheme in her theorizing that allows her to identify the possibility of determining the appropriate context for identifying the emergent properties of each type, and this is also what we have established in the present study. Furthermore, the objective with which we have used this approach here is to proceed toward an integration of all those elements necessary to observe the change in the meaning of a hashtag.

Compared to the first limit set out in this study, we will need to verify through research if it can be empirically tested; although the studies in which text mining and topic modeling are applied already signal a gradual change in the topics of discussion. In this sense, the formalization of this model can help the researcher to focus on this cultural change through the meanings built around a theme/event. The practical application of this model in the research would represent the welding phase of the graft we have discussed in this paper.

## Author contributions

Both authors listed have made a substantial, direct, and intellectual contribution to the work and approved it for publication.
